# Thromboprophylaxis in congenital nephrotic syndrome: 15-year experience from a national cohort

**DOI:** 10.1007/s00467-020-04793-z

**Published:** 2020-10-21

**Authors:** Laurence J. Dobbie, Angela Lamb, Lucy Eskell, Ian J. Ramage, Ben C. Reynolds

**Affiliations:** 1 University of Glasgow, Glasgow, UK; 2 Department of Paediatric Nephrology, Royal Hospital for Children, 1345 Govan Road, Glasgow, G51 4TF UK

**Keywords:** Infantile nephrotic, Warfarin, Low molecular weight heparin, Venous thromboembolism, Anticoagulation

## Abstract

**Introduction:**

Congenital nephrotic syndrome (CNS) is an ultra-rare disease associated with a pro-thrombotic state and venous thromboembolisms (VTE). There is very limited evidence evaluating thromboprophylaxis in patients with CNS. This study aimed to determine the doses and duration of treatment required to achieve adequate thromboprophylaxis in patients with CNS.

**Methods:**

From 2005 to 2018 children in Scotland with a confirmed genetic or histological diagnosis of CNS were included if commenced on thromboprophylaxis. The primary study endpoint was stable drug monitoring. Secondary outcomes included VTE or significant haemorrhage.

**Results:**

Eight patients were included; all initially were commenced on low-molecular weight heparin (enoxaparin). Four patients maintained therapeutic anti-Factor Xa levels (time 3–26 weeks, dose 3.2–5.07 mg/kg/day), and one patient developed a thrombosis (Anti-Factor Xa: 0.27 IU/ml). Four patients were subsequently treated with warfarin. Two patients maintained therapeutic INRs (time 6–11 weeks, dose 0.22–0.25 mg/kg/day), and one patient had two bleeding events (Bleed 1: INR 6, Bleed 2: INR 5.5).

**Conclusions:**

Achieving thromboprophylaxis in CNS is challenging. Similar numbers of patients achieved stable anticoagulation on warfarin and enoxaparin. Enoxaparin dosing was nearly double the recommended starting doses for secondary thromboprophylaxis. Bleeding events were all associated with supra-therapeutic anticoagulation.

**Electronic supplementary material:**

The online version of this article (10.1007/s00467-020-04793-z) contains supplementary material, which is available to authorized users.

## Introduction

Congenital nephrotic syndrome (CNS) is a rare disease characterised by heavy proteinuria and severe oedema developing within 3 months of birth [1, 2]. Glomerular filtration barrier proteins are defective due to genetic mutations or more rarely secondary to congenital viral infection. Complications arising from severe proteinuria include venous thromboembolism (VTE), recurrent infection, fluid and electrolyte disturbance, and impaired growth [3]. The increased VTE risk is predominantly attributed to urinary loss of proteins important in coagulation regulation, exacerbated by the common requirement in this patient group for long-term central venous access [4–6]. Loss of haemostatic proteins, e.g., antithrombin III, leads to an up-regulation in hepatic coagulation factor synthesis and thus a pro-thrombotic tendency [7–10]. Several studies report a VTE prevalence of 10–29% of CNS patients over their disease course; this variability being partly attributed to the marked genotypic and phenotypic variation in CNS [1, 11, 12].

To mitigate the thrombotic risk, management includes strategies to reduce urinary protein loss and administration of anticoagulant therapies. Protein loss is minimised by bilateral nephrectomy and early use of dialysis, or unilateral nephrectomy in combination with angiotensin converting enzyme inhibitors and prostaglandin inhibitors to decrease GFR [4, 13]. Anticoagulation agents commonly used are warfarin and enoxaparin. Warfarin, a vitamin K antagonist, is monitored using the international normalised ratio (INR). The target INR is between 2.0 and 3.0 for primary thromboprophylaxis [14]. Enoxaparin, a low molecular weight heparin (LMWH), binds to anti-thrombin leading to inhibition of activated factor X. Anti-factor Xa assays are used to monitor efficacy, with a target level between 0.2 and 0.4 IU/ml for primary thromboprophylaxis [14, 15]. If a thrombotic event has already occurred, levels are targeted at 0.5–1 IU/ml for secondary thromboprophylaxis. Aspirin is less frequently used as thromboprophylaxis in CNS and is not utilised within our unit. Unfractionated heparin is not suitable as it requires continuous infusion, as well as an extensive adverse effect profile [2]. Direct oral anticoagulants have not been studied in CNS.

Thromboprophylaxis in children is challenging due to rapid growth velocity and physiological changes in pharmacokinetics, especially in the early years of life [16, 17]. Fung et al. demonstrated that therapeutic anti-factor Xa levels required an average of 1.64 mg/kg and 1.45 mg/kg of enoxaparin for children under 1 year and aged 1 to 6 years, respectively [16, 18]. Thromboprophylaxis using LMWH in CNS is further complicated by antithrombin III deficiency (due to urinary loss) causing heparin resistance [19]. Warfarin also has challenges in infancy, as metabolism is influenced by comorbidities, medications, and dietary changes. Similar to enoxaparin, higher doses are typically required in infants than children with doses of ~ 0.32 mg/kg and ~ 0.09 mg/kg reported in children under 1 and over 11, respectively [20]. Infants also typically require longer treatments to achieve target INRs and more frequent dose adjustments when compared with older children [21].

The extreme rarity of CNS is a significant limitation on the ability to undertake a clinical trial of thromboprophylaxis. Therapeutic decisions are based on patient preference and clinician experience. In a recent European multi-centre retrospective review of anticoagulation in CNS, 5/45 (11%) patients receiving anticoagulant therapy and 4/26 (15%) not receiving anticoagulants developed VTE (*p* = 0.60) [22]. Anticoagulant therapies in patients experiencing VTE were warfarin (*n* = 3), heparin (*n* = 1), and aspirin (*n* = 1). Despite participation by 17 tertiary centres, the rarity of CNS and VTE as an outcome precluded formal statistical analysis due to small numbers. Additionally, therapeutic monitoring was not reported, making it uncertain whether VTE occurred due to inadequate thromboprophylaxis in the ‘anticoagulated’ cohort. Our own observation was that patients often required high doses of anticoagulant agents to achieve sufficient therapeutic levels. This case series aims to report whether significantly higher doses of anticoagulants are required to achieve adequate thromboprophylaxis in patients with CNS. We hypothesised that patients will require high doses of anticoagulants with a prolonged time taken to reach therapeutic levels.

## Methods

Data were obtained from patients admitted to the Royal Hospital for Children, Glasgow. Patients were included if CNS was diagnosed from 1 July 2005 until 1 January 2018. The database was locked on 1 June 2020. As a single national paediatric nephrology centre, this represents all CNS cases in Scotland in that time period. The data were collected retrospectively using clinical portal (TrakCare, InterSystems corporation) and the Strathclyde electronic renal patient record (SERPR) (VitalDataClient, v1.6.0.9493). Graphs were produced using GraphPad Prism version 8 (GraphPad Software, San Diego, CA).

Data collected included basic demographic data, length, weight, serum creatinine, serum albumin, urinary protein:creatinine ratio, factor Xa assays, INR, antithrombin III levels, thromboprophylaxis dose in mg/kg/day, concomitant medications, albumin infusion data, genetic analyses (where performed), any confirmed thrombo-embolic events, and any confirmed haemorrhagic events (both determined by clinical discussion).

Estimated glomerular filtration rate (eGFR) was calculated using the Bedside IDMS-traceable Schwartz GFR equation (GFR (ml/min/1.73 m^2^) = (36.2 × length (cm))/creatinine (μmol/l)). In cases where length data was unavailable early in clinical course (*n* = 3), growth chart values were extrapolated backwards along their centile to provide an estimate of length at the time of presentation.

The primary study endpoint was effective and stable thromboprophylaxis, defined as three consecutive therapeutic measurements. Therapeutic levels of enoxaparin were defined as anti-factor Xa levels of 0.2–0.4 IU/ml; therapeutic warfarinisation was defined as INR between 2.0 and 3.0. In patients where a thrombotic event occurred prior to anticoagulation, secondary thromboprophylaxis levels were targeted to anti-factor Xa levels of 0.5–1.0 IU/ml. Secondary endpoints were bilateral nephrectomies, transplantation, or the development of stage 5 chronic kidney disease (CKD 5), defined as confirmed eGFR < 15 ml/min/1.73 m^2^ (i.e., the value was calculated using a measured height, not via extrapolation). Where patients switched thromboprophylaxis modality, data were also collected from the onset of the second therapy, until the same endpoint was reached. Secondary outcomes included clinically confirmed VTE or any clinically significant episode of haemorrhage.

## Results

Eleven children had a confirmed diagnosis of CNS between 1 July 2005 and 1 January 2018. Three children were not included. One child died at 2 weeks of age, one presented initially with severe acute kidney injury requiring haemofiltration and had a persistent requirement for dialysis thereafter for fluid removal (patient 9), and the third was in CKD 5 at the time of presentation (patient 10). Table 1 summarises the relevant demographic, phenotypic, and clinical details of all included patients. Supplementary Table 1 summarises excluded patients. There were five male patients and three female, with clinical presentation at a mean age of 6 weeks (range 2–15 weeks). Clinically, one patient had Pierson syndrome and two had Denys Drash syndrome. Histologically, four patients had diffuse mesangial sclerosis, two patients had ‘stage 5’ histological findings, one patient had mild glomerular change only, and one patient had no biopsy undertaken. Mutational analysis showed that five patients had mutations affecting *NPHS1*, one had a *LAMB2* mutation, and two had *WT1* mutations. Table 2 details the mutational analyses in patients where available. The eGFR at presentation was highly variable between patients (range 16–177 ml/min/1.73 m^2^) as was presenting serum albumin (range 6–21 g/L). Proteinuria data was available for 5/8 patients at presentation (range 3.81–9.63 g/mmol). Antithrombin III levels were measured in 2 patients at presentation, both below the normal range (patients: 25–61 IU/dL, normal: 71–101 IU/dL). Measurement of antithrombin III is not routine in our institution, and no other results at presentation were available.

**Table 1:** Demographic and clinical summaries of all included patients

Patient	1	2	3	4	5	6	7	8
Sex	M	M	M	M	M	F	F	F
Associated phenotypic syndrome	None	None	None	None	None	Denys Drash	Pierson	Denys Drash
Histology	50–80% global glomerulosclerosis, increased mesangial matrix, chronic interstitial inflammation, proximal tubular dilatation	80% global glomerulosclerosis, increased mesangial matrix, chronic interstitial inflammation, cystic tubular dilatation, marked interstitial fibrosis/tubular atrophy	DMS	10% global glomerulosclerosis, 50% minor glomerular synechiae. Predominantly normal tubules. V mild interstitial fibrosis	DMS	DMS	Not done	DMS
Genetic mutation (Table 2)	*NPHS1* homz	*NPHS1* comHet	*NPHS1* comHet	*NPHS1* comHet	*NPHS1* comHet	*WT1*	*LAMB2*	*WT1*
Age at presentation (weeks)	3	2	2	9	4	15	7	2
Initial eGFR (ml/min/1.73 m^2^)	72	177	145	149	151	64	40	16
Initial Serum albumin (g/L)	11	10	6	10	6	13	21	6
Initial antithrombin III level (IU/dL) (normal 71-101)	NM	NM	NM	NM	NM	25	61	NM
Initial uPCR (g/mmol)	NM	NM	8.10	NM	3.81	6.96	8.83	9.63
Enoxaparin primary end point	Never therapeutic, discontinued after 25 weeks	6 weeks to therapeutic	Therapeutic at 6 weeks	Never therapeutic after 27 weeks	Therapeutic at 26 weeks	CKD 5 at 10 weeks	CKD 5 at 9 weeks	Therapeutic at 3 weeks
Warfarin primary end point	11 weeks to therapeutic	6 weeks to therapeutic	N/A	Never therapeutic after 50 weeks therapy	Discontinued after 22 weeks due to bleeding concerns	N/A	N/A	N/A
Outcome	Transplant aged 6 years	Transplant aged 4 years	Deceased (05/2020)—unknown cause	Spontaneous improvement, now CKD3 aged 14 years	Unilateral Nephrectomy Deceased aged 3 years	Deceased aged 3 years	Deceased aged 6 months	Bilateral nephrectomy (06/2018), on PD

*Homz* homozygous, *comHet* compound heterozygote, *eGFR* estimated glomerular filtration rate, *uPCR* urinary protein creatinine ratio, *M* male, *F* female, *NPHS1* nephrin, *LAMB2* beta-2-laminin, *CKD 5* stage 5 chronic kidney disease, *DMS* diffuse mesangial sclerosis, *NM* not measured, *PD* peritoneal dialysis

**Table 2:** Complete mutational analyses for all patients

Patient	Genetics
1	*NPHS1*: Homozygous mutation c.2417c > G Highly likely to be pathogenic
2	*NPHS1*: Compound heterozygote c.523C > T exon 5, nonsense c.1379G > A exon 11, missense Both highly likely pathogenic
3	*NPHS1*: Compound heterozygote c.1954C > T exon 15, nonsense c.2335-1G > A intron 17, skip/frameshift Likely pathogenic and highly likely pathogenic respectively
4	*NPHS1*: Compound heterozygote c.2335-1G > A intron 17 – skip/frameshift c.2491C>T exon 18 missense Highly likely pathogenic and likely pathogenic respectively
5	*NPHS1*: Compound heterozygote c.2227C > T exon 17 – missense c.2335-1G > A intron 17 – skip/frameshift Both classed highly likely pathogenic
6	*WT1*: Heterozygous c.[443-6C>A];[=] Classed as unlikely pathogenic
7	*LAMB2*: Homozygous splice site variant in intron 25 c.3982 + 1G > T Pathogenic, unknown effect but predicted to skip exon 25
8	*WT1*: De novo novel heterozygous frameshift variant on exon 9 c.[1201delA];[1202=] Likely pathogenic.
9	*LAMB2*: Homozygous c.736C > T exon 7 – missense Pathogenic
10	*WT1*: Heterozygous c.1181G > A exon 9 – missense

*NPHS1* nephrin, *LAMB2* beta-2-laminin, *WT1* Wilms tumour 1

All patients had a central venous catheter (CVC) inserted for either the delivery of intravenous albumin or the provision of haemodialysis. The albumin requirement varied from 6.3 to 31.5 g/kg/week. Further detail on albumin requirements are provided in Supplementary Table 2. Standard medical management in our unit also included regular administration of phenoxymethylpenicillin (penicillin V), levothyroxine as needed, angiotensin-converting enzyme inhibition (ACEi), and anti-reflux medications.

### Enoxaparin dosing

All included patients were commenced on LMWH (enoxaparin) as a first-line thromboprophylaxis agent, at a mean starting dose of 1.88 mg/kg/day (range 0.71–4.3 mg/kg/day). The dose then subsequently varied from 0.71 mg/kg/day to a maximum of 7.44 mg/kg/day. All patients received subcutaneous administration twice a day with anti-factor Xa levels measured at 4 to 6 h post-dose. No patients received enoxaparin via infusion. Antithrombin III levels were not routinely measured, though 3 patients had at least one measurement (always below normal). No patient received antithrombin III infusions.

**Fig. 1:**
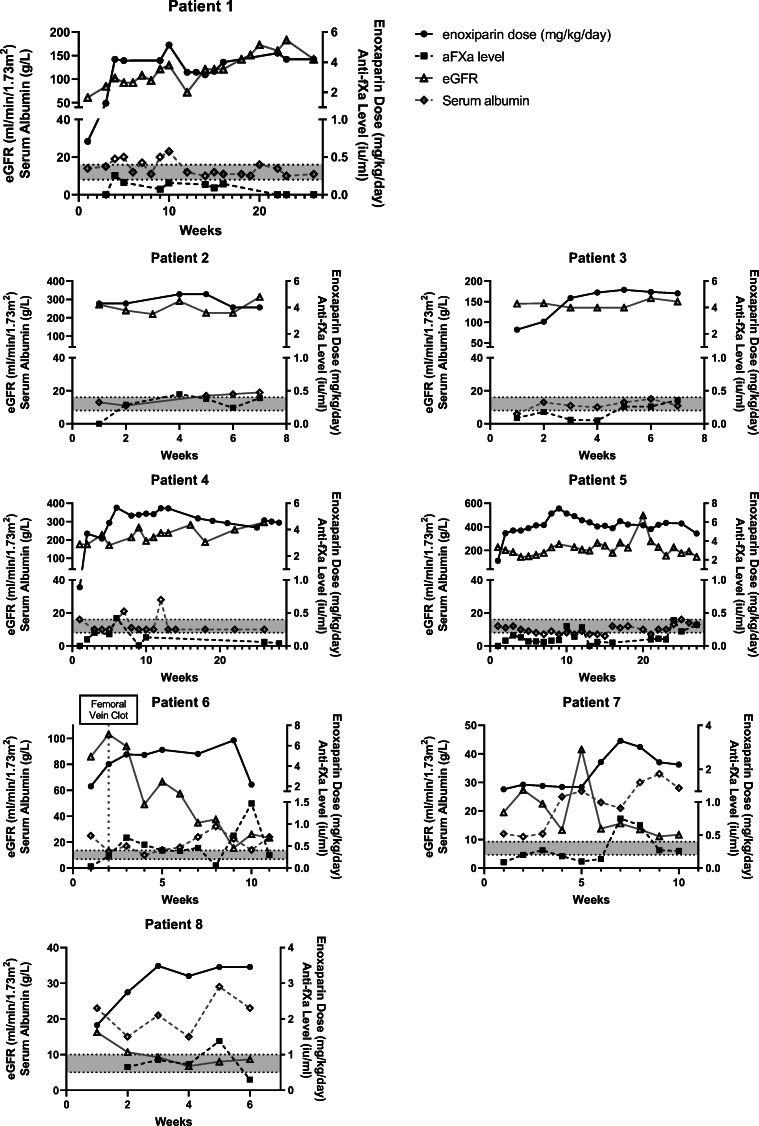
Enoxaparin data. Graphs demonstrating individual patient enoxaparin dosing, therapeutic monitoring using anti-factor Xa, eGFR, and serum albumin. The left *y*-axis displays eGFR and serum albumin data; the right *y*-axis displays enoxaparin dose and anti-factor Xa level. The grey shaded area represents the target therapeutic range for thromboprophylaxis. The vertical grey dotted line represents an adverse event

Figure 1 details graphs of enoxaparin dosing, anti-factor Xa levels, eGFR, and serum albumin (Supplementary Figure 1 replaces serum albumin with urinary protein:creatinine ratio where available). Four patients reached therapeutic anti-factor Xa levels with the dose varying from 3.2 to 5.07 mg/kg/day. and time taken varying from 3 to 28 weeks (Table 1; patient 2 and 3: 6 weeks, 4.0 mg/kg/day and 5.07 mg/kg/day, respectively; patient 5: 26 weeks, 4.79 mg/kg/day; patient 8: 3 weeks, 1.82 mg/kg/day). Four patients did not reach therapeutic anti-factor Xa levels. Two patients reached CKD 5 before therapeutic levels were achieved, resulting in discontinuation of anticoagulation. Two patients had discontinuation due to failure to achieve adequate levels despite dose escalation, occurring after 25–27 weeks of therapy. The patients achieving therapeutic LMWH levels had *NPHS1* compound heterozygote or *WT1* mutations (patients 2, 3, and 5 = *NPHS1* compound heterozygote, patient 8 = *WT1* mutation). An apparent inverse relationship was noted between eGFR and anti-factor Xa levels, i.e., a decrease in eGFR associated with an increase in anti-factor Xa levels as might be physiologically expected. Serum albumin was proportional, with a higher serum albumin associated with higher anti-factor Xa levels.

### Warfarin dosing

Four patients were subsequently commenced on warfarin, at a mean starting dose of 0.19 mg/kg/day (range 0.18–0.2 mg/kg/day). The dose then varied from 0.18 mg/kg/day to a maximum of 0.89 mg/kg/day.

**Fig. 2:**
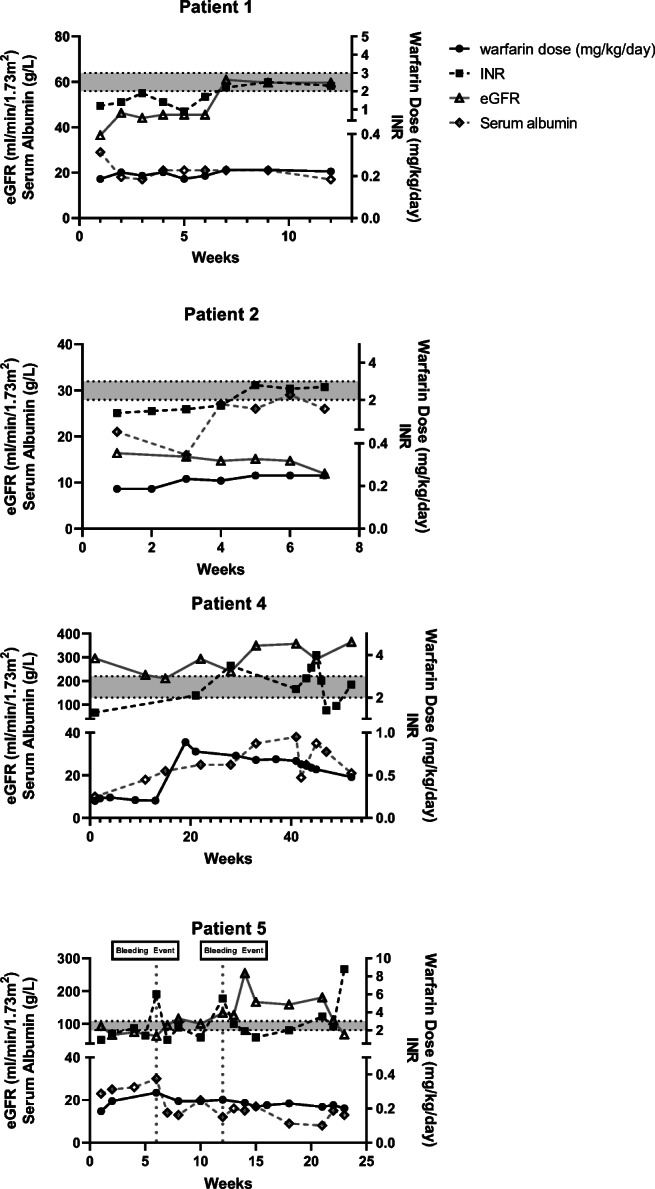
Warfarin data. Graphs demonstrating individual patient warfarin dosing, therapeutic monitoring using INR, eGFR, and serum albumin. The left *y*-axis displays eGFR and serum albumin data; the right *y*-axis displays warfarin dose and INR. The grey shaded area represents the target therapeutic range for thromboprophylaxis. The vertical grey dotted line represents an adverse event

Figure 2 details graphs of warfarin dosing, INR, eGFR and serum albumin (Supplementary Figure 2 replaces serum albumin with uPCR for patient 5). Two patients reached therapeutic INRs with doses from 0.22 to 0.25 mg/kg/day and time taken varying from 6 to 11 weeks (Table 1; patient 1: 11 weeks, 0.22 mg/kg/day; patient 2: 6 weeks, 0.25 mg/kg/day). Two patients did not reach therapeutic INR. Patient 4 did not reach therapeutic levels after 1 year and patient 5 was discontinued from warfarin after 22 weeks due to concerns regarding bleeding. For eGFR and INR the graphs again show an inverse relationship.

Supplementary figure 3 provides similar information for non-included patients 9 and 10.

### Adverse events

Tables 3 and 4 summarise identified adverse events in included patients (clinical vignette 1 provides the same for patient 9). Relevant kidney parameters and anticoagulation data at the time are included. Supplementary Table 3 details concomitant medications at the time of adverse events. There were two bleeding events and one thrombotic event during follow-up. One thrombotic event occurred prior to thromboprophylaxis in this cohort.

**Table 3:** Anticoagulation and complication data for all included patients

Patient	1st drug	Starting dose (minimum-maximum) (mg/kg/day)	Dose when therapeutic (mg/kg/day)	Time to therapeutic dose	eGFR start	eGFR when therapeutic	2nd drug	Starting dose (minimum–maximum) (mg/kg/day)	Dose when therapeutic	Time to therapeutic dose	eGFR start	eGFR when therapeutic	Thrombus	Bleeding
1	Enoxaparin	0.71 (0.71-5.14)	N/A	Never therapeutic	60.8	N/A	Warfarin	0.19 (0.19–0.23)	0.22	11 weeks	36.4	59.6	N/A	N/A
2	Enoxaparin	4.3 (2.9–5)	4.0	6 weeks	271.5	313.2	Warfarin	0.19 (0.19–0.25)	0.25	6 weeks	16.4	11.9	N/A	N/A
3	Enoxaparin	2.3 (2.3-5.78)	5.07	6 weeks	145	150	N/A	N/A	N/A	N/A	N/A	N/A	N/A	N/A
4	Enoxaparin	0.89 (0.89–5.62)	N/A	Never therapeutic	176.1	N/A	Warfarin	0.2 (0.2–0.89)	N/A	Never therapeutic	295.5	N/A	N/A	N/A
5	Enoxaparin	1.9 (1.9–7.44)	4.79	26 weeks	226.25	145.9	Warfarin	0.18 (0.18–0.25)	N/A	Never therapeutic	93.1	N/A	N/A	2 Bleeding events
6	Enoxaparin	2 (2–6.53)	N/A	Never Therapeutic	85.98	N/A	N/A	N/A	N/A	N/A	N/A	N/A	Right femoral vein thrombus	N/A
7	Enoxaparin	1.1 (1.1–6)	N/A	Never therapeutic	19.5	N/A	N/A	N/A	N/A	N/A	N/A	N/A	N/A	N/A
8	Enoxaparin	1.82 (1.82–3.48]	3.2	3 weeks	16.25	6.8	N/A	N/A	N/A	N/A	N/A	N/A	SVC thrombus pre-thromboprophylaxis	N/A

*eGFR* estimated glomerular filtration rate, *N/A* not applicable

**Table 4:** Thrombotic and bleeding events and relevant parameters

Patient	Adverse event	Age at event (weeks)	Drug	Time to event from starting medication (weeks)	Dose (mg/kg/day)	INR	Anti-factor Xa level (IU/ml)	eGFR (ml/min/1.73 m^2^)	Serum albumin (g/L)	Platelets (x 10^9^/L)	uPCR (g/mmol)	Additional data
5	Bleeding	50	Warfarin	5	0.293	6	N/A	63.4	30	174	10.36	Blood altered vomiting and stools with infection in PEG
5	Bleeding	56	Warfarin	11	0.252	5.5	N/A	133.1	12	274	Nil	Haematemesis with 1 week history of viral infection. Blood dried around gastrostomy site.
6	Thrombus – femoral vein	17	Enoxaparin	1	4.19	N/A	0.27	103.2	13	454	41.72	Haemodialysis dependent, low iron, hypothyroidism.
8	Thrombus – SVC	2	N/A	N/A	N/A	N/A	N/A	8	16	373	9.63	Managed in PICU, treated for maternal Grave’s disease

*eGFR* estimated glomerular filtration rate, *INR* international normalised ratio, *N/A* not applicable

### Bleeding

Patient 5 had two bleeding events after 5 and 11 weeks of therapy, both whilst on warfarin. This coincided with a supratherapeutic INR. The patient was haemodynamically stable on both occasions. The first bleeding event occurred 3 months following unilateral nephrectomy, whilst on home IV albumin. The patient presented with fresh red blood evident in the stool, with visible clot. The patient’s gastrostomy was noted to be leaking with evidence of superficial infection. Indomethacin was temporarily discontinued, IV omeprazole administered, and warfarin withheld. The INR was 6. Packed red cells were transfused to improve haemoglobin (pre-transfusion, 54 g/L). Twelve hours post-presentation, there was fresh blood leakage from the gastrostomy, coinciding with coffee-ground vomiting. IV vitamin K was administered at a dose of 30 mg/kg to reverse over-warfarinisation without preventing ongoing thromboprophylaxis. Warfarin was withheld for 48 h then re-commenced at the original dose.

The second bleeding event occurred 1 week following an upper respiratory tract infection, 1 month after the initial bleeding event, presenting again with blood-specked vomitus and fresh blood leakage from the gastrostomy. Haemoglobin had fallen from 99 to 70 g/L. INR was ‘unrecordable’ twice, so IV vitamin K was administered, again at 30 mg/kg. Repeat INR 6 h later was 5.5. Transfusion was not required on this occasion. Warfarin was recommenced at a slightly lower dose after 72 h.

Two months later, the same patient then had an incidental finding of an INR of 8.8 with no associated bleeding symptoms. At that point, warfarin was discontinued and the patient re-commenced on LMWH.

### Thrombus

No thrombotic complications developed whilst patients were adequately warfarinised.

Patient 6 had identification of a femoral vein thrombus aged 4 months, 2 weeks following initial presentation. Initial management required continuous veno-venous haemofiltration (CVVH) initially via a femoral CVC, which was changed to a left internal jugular CVC 3 days into therapy. CVVH was discontinued after 4 days, and the patient was commenced on enoxaparin. One week later, the patient developed evident discrepancy in leg size, with identification of non-occlusive thrombus within the right femoral vein. This coincided with a thromboprophylactic anti-factor Xa level of 0.27 IU/ml. At the time of thrombus detection, the patient was proteinuric (uPCR of 41.72 g/mmol), hypoalbuminaemic (13 g/L), and had a mild thrombocytosis (454 × 10^9^/L). Following detection of the thrombus, the target anti-factor Xa was temporarily increased to 0.5–1.0 IU/ml until the clot resolved, and for 3 months subsequently.

Patient 8 developed a superior vena cava (SVC) thrombus 5 days following initial insertion of an internal jugular CVC at 2 weeks of age, prior to the commencement of anticoagulation. Enoxaparin was subsequently initiated as secondary thromboprophylaxis, with target levels of 0.5–1.0 IU/ml. Of note, the patients’ mother also had Grave’s disease, which may have further exacerbated thrombosis risk.

At the time of database lock, two patients had successfully been transplanted, four patients had died (cause of mortality: sepsis = 1, cardiomyopathy = 1, intestinal obstruction and perforation = 1, probable autonomic failure = 1), one patient was on peritoneal dialysis, and one had ongoing CKD stage 3.

## Discussion

This case series describes the challenges in achieving effective and safe thromboprophylaxis in patients with CNS. Enoxaparin led to adequate thromboprophylaxis in 4/8 patients compared with 2/4 patients on warfarin, with variable therapeutic times and doses. Both agents had similar safety profiles. All bleeding complications were associated with supra-therapeutic measurements, highlighting the requirement for careful monitoring. Anti-factor Xa levels and INR appear to have an inverse relationship with kidney function, as might be physiologically expected. Loss of kidney function reduces proteinuric losses of antithrombin III and other relevant proteins, which may contribute to more effective anticoagulation.

The British National Formulary for children (BNFc) is the standard formulary within the UK and recommends an initial enoxaparin dose of 1 mg/kg/day for secondary thromboprophylaxis for children aged over 2 months (an initial dose of 2 mg/kg/day is recommended under 2 months, due to differences in infant drug handling) [23]. International guidelines suggest higher doses for younger children [14]. Our study cohort all received higher doses than BNFc guidelines, both initially and once therapeutic. The mean initial dose in our cohort was 1.88 mg/kg/day, nearly double the recommended starting dose, with the therapeutic dose ranging from 3.2 to 5.07 mg/kg/day. The mean enoxaparin dose required to achieve adequate primary thromboprophylaxis was 4.27 mg/kg/day, over 4 times the suggested dose. The requirement for higher doses may be attributable to a generally younger age, lower antithrombin III levels related to proteinuric loss (below the normal range in all patients where measurement was performed; Table 1), and potentially other relevant urinary losses [14, 18]. Dosing variability likely also reflects the genotypic and phenotypic differences within our small cohort, including the degree of proteinuria. Though therapeutic monitoring is not generally undertaken in adults on enoxaparin, the volatile nature of both proteinuria and kidney function mandates monitoring in paediatric patients. All patients in this cohort had administration of enoxaparin twice daily, though once daily dosing is also described. Though there are no reported differences in safety or efficacy between a once or twice daily dosing regimen, the available pharmacokinetic data supports a twice daily dosing regimen [24, 25].

As expected, warfarin dosing was variable between patients and required careful titration and monitoring, similar to other patient groups. Our cohort’s mean initial dose was 0.19 mg/kg, similar to the recommended initial dose of 0.2 mg/kg. Our cohort reflects the known literature, with warfarin dosing ranging from 0.18 to 0.89 mg/kg, and a mean dose of 0.24 mg/kg achieving an INR suitable for primary thromboprophylaxis. In one prospective study, infants required higher doses of warfarin than older children, with infants under 1 requiring ~ 0.32 mg/kg, whereas children over 11 years required ~ 0.09 mg/kg [20]. Patient 4 never reached a therapeutic INR despite dose escalation to 0.89 mg/kg. Warfarinisation of children is challenging, even more so in patients with ongoing alterations in their haematologic physiology [16, 21].

To our knowledge this is the first study to address and report actual monitoring of thromboprophylaxis in a national cohort of CNS patients. A recent multi-centre retrospective review of anti-thrombotic prophylaxis was carried out in 17 centres over 15 European countries. The investigators reported that 4/45 (11%) receiving anticoagulants and 5/26 (15%) not receiving anticoagulants developed VTEs (*p* = 0.60). Notably, the majority of VTEs in that cohort occurred whilst patients were warfarinised (warfarin in 3, heparin in 1, aspirin in 1). This finding contrasts with our observation of VTEs only occurring in a heparinised patient, though our cohort is both smaller and has a different genetic mix (69% *NPHS1* and 14% *WT1* in Dufek et al., 50% and 25% respectively for our cohort) [22]. A separate retrospective review of anticoagulated CNS patients reported a VTE rate of 29% (16/55). About 67% (37/55) of that cohort had an *NPHS1* mutation, and no patients had a *LAMB2* mutation—unlike the 2/8 in our cohort [11]. Our cohort has a relatively high prevalence of non-*NPHS1* mutations or novel *NPHS1* mutations, which may limit the comparability and generalisation of our results. Neither of the two larger studies reported assays indicating effective thromboprophylaxis, or whether dosing and kidney function influenced anticoagulant efficacy.

Two further retrospective studies have investigated prophylactic anticoagulation in adults with nephrotic syndrome (NS). A Danish retrospective analysis investigated 79 patients; of whom 44 were anticoagulated and 35 were not and reported a significant reduction in thrombotic events (4 versus 0 episodes, *p* = 0.035) in patients receiving anticoagulant therapy without increasing bleeding episodes (*p* = 0.45) [26]. A second retrospective study reported thrombotic events in 1.39% (2/143) of anticoagulated patients and concluded that anticoagulation effectively reduced the VTE rate in nephrotic syndrome which reportedly ranges from 7 to 40% [27]. Though the adult NS literature suggests a role for thromboprophylaxis in reducing the VTE risk, the aetiology of adult NS is very different, even to idiopathic childhood NS, which is a further separate clinicopathological entity to CNS, including the degree of proteinuria which is typically many fold higher in CNS than idiopathic NS. Extrapolating findings from adult studies to this patient cohort must be done with caution.

Within our cohort, only 50% (4/8) of heparinised and 50% (2/4) of warfarinised patients achieved adequate thromboprophylactic levels prior to the onset of CKD 5. Bleeding events occurred in 1 of 4 warfarinised patients. The only thrombosis on treatment developed with enoxaparin at an adequate thromboprophylactic level. The small sample size precludes formal analysis or recommending one agent over another. All patients were initially heparinised, with warfarin used as second-line thromboprophylaxis in our unit. It is plausible that adequate thromboprophylaxis is more readily achieved later in the disease course, due to patients being more stable, or having reduced overall proteinuric loss. A larger cohort of patients receiving either warfarin or enoxaparin initially would be required to truly determine the more efficacious agent. For reasons previously described, this is unlikely to occur.

Patient 7 required a significantly lower dose of enoxaparin to reach target anti-factor Xa levels. This could be partly explained by the patient’s early development of significant CKD and lesser degree of proteinuria. This patient also represents the only included patient with *LAMB2* mutation, again indicating genotypic variability.

All patients had CVCs. This is an established risk factor for the development of VTEs; in one reported cohort ~ 5% of paediatric patients with CVCs in situ had at least one VTE [28]. In both cases of thrombus in this cohort (patient 6 and 8), thrombus was detected within a catheterised or recently catheterised vessel, and within 2 weeks of initial presentation. As a CVC is often fundamental to CNS management, risk mitigation can only be via timely thromboprophylaxis. Using higher than BNFc recommended initial dosing may achieve this, though that conclusion cannot be drawn from our cohort [14].

Warfarin has many potential medication interactions which could have prevented target INRs. All warfarinised patients were prescribed antibiotics concurrently which could have altered warfarin’s pharmacodynamics. Additionally, patient 5 developed a central line sepsis and thrombocytopenia. This could partly explain why this patient had repeated bleeding events coinciding with supraphysiological INRs. Yet, in this patient population there are likely to be many unavoidable confounders to therapeutic warfarinisation due to the complexities of CNS management.

Though multiple medications can potentiate or inhibit the actions of thromboprophylaxis, the doses of concomitant medications used routinely in these patients (e.g. antibiotic prophylaxis) were typically standard and infrequently altered. The effect on thromboprophylaxis pharmacokinetics would therefore be consistent and unlikely to account for sudden changes in INR or anti-factor Xa. These patients are complex with multiple factors impacting on both pharmacokinetics and pharmacodynamics—further supporting the need for regular therapeutic surveillance.

The management of CNS typically includes regular infusions of IV albumin, the dose of which reflects the degree of proteinuria. Weekly albumin doses varied within the cohort from 5 to 32 g/kg/week (Supplementary Table 2). There was no apparent association between dose of albumin administered and likelihood of achieving adequate thromboprophylaxis. Patient 4 in this cohort never required IV albumin, and had a different clinical course, similar to that seen in Maori populations. Yet this patient was the most difficult patient to manage thrombotic risk, failing both LMWH and warfarin despite prolonged treatment with both [1].

Two patients had a long period of sub-therapeutic treatment of enoxaparin with minimal dosing changes (Fig. 1: patient 1: 25 weeks, patient 2: 27 weeks). Prolonged sub-therapeutic therapy could increase the VTE risk, necessitating consideration of conversion to warfarin. Achieving effective thromboprophylaxis for these patients was challenging, as in some eGFR increased with time, possibly resulting in elevated clotting factor excretion. Clinical instability may cause clinicians to be reluctant to alter medication dosage, which may partly explain the long sub-therapeutic period. Conversely, one warfarinised patient was converted back to enoxaparin due to safety concerns from unstable and excessive INR, and two episodes of gastrointestinal bleeding.

The cohort is from a single national centre with 100% patient identification over a 15-year period, with all patients treated by the same clinical team thereby reducing variability in clinical treatment.

This dataset is (to our knowledge) unique in showing the relationship between anticoagulant dosing, therapeutic drug levels, and kidney function in patients with CNS. The optimal therapeutic regimen in this patient population has not been ascertained. Though our cohort is too small to definitively comment on dosing regimen or choice of thromboprophylaxis, the safety profiles confirm the importance of measuring therapeutic levels regularly in this complex patient group.

There are limitations to this cohort. The patient group were heterogeneous, histologically and genetically, which may have conferred different risk profiles of VTE [27]. The variability in clinical course affecting both proteinuria and kidney function will also have an impact on interpretation. This heterogeneity further highlights the difficulties in establishing an evidence base for thromboprophylaxis in CNS.

The small sample size precludes statistical analysis, unavoidable due to the disease rarity. A sufficiently large cohort would mandate further international trials, but the most recent effort demonstrated how challenging this is. Despite engaging 22 tertiary European centres, that study failed to recruit enough patients to achieve statistical power for outcomes [22].

The limited data on proteinuria prevents interrogation of the relationship between therapeutic drug levels and urinary protein. Retrospective review of healthcare records for outcome reporting is recognised to have flaws, as minor but clinically relevant episodes may not be reported or poorly documented. This is somewhat mitigated by the lengthy in-patient stays of these patients. All adverse events have occurred in a hospital setting.

For three patients (4–6) length data was unavailable in the early parts of life, so eGFR was calculated by retrospective extrapolation using the patient’s nearest available length centile. This may overestimate earlier length as early management of CNS includes optimising nutrition and growth. To limit the impact of this, the outcome of CKD 5 was only assigned when using either a confirmed patient length, or where kidney replacement therapy was required. It is plausible that early kidney function was overestimated for those patients.

## Conclusions

This case series demonstrates that achieving adequate and stable thromboprophylaxis in children with CNS is challenging. All bleeding events were associated with supra-therapeutic levels. Development of thrombus prior to or shortly after any thromboprophylaxis highlights the importance of commencing this early. Enoxaparin doses required for thromboprophylaxis in this patient population were approximately double the recommended dose.

## Electronic supplementary materials

ESM 1 (DOCX 233 kb).

## Data Availability

The datasets generated and analysed during the current study are available from the corresponding author on reasonable request.

## Abbreviations

BNFc: British National Formulary for Children
CNS: Congenital Nephrotic Syndrome
CVVH: Continuous veno-venous hemofiltration
eGFR: Estimated glomerular filtration rate
INR: International Normalised Ratio
LMWH: Low molecular weight heparin
SVC: Superior vena cava
VTE: Venous Thromboembolism
UPCR: Urinary protein:creatinine ratio
