# Supplementary material for: Thromboprophylaxis in congenital nephrotic syndrome: 15-year experience from a national cohort
Source: Pediatr Nephrol. 2020 Oct 21;36(5):1183–94. doi: 10.1007/s00467-020-04793-z (PMC8009789; doi:10.1007/s00467-020-04793-z)
Supplement: Supplementary file 1 — (DOCX 233 kb). [file 467_2020_4793_MOESM1_ESM.docx]

Supplementary Material for:

Thromboprophylaxis in Congenital Nephrotic Syndrome: 15 year experience from a national cohort.

Laurence J. Dobbie^1^, Angela Lamb^2^, Lucy Eskell^2^, Ian J Ramage^1,2^, Ben C Reynolds^1,2^

1. University of Glasgow
2. Department of Paediatric Nephrology, Royal Hospital for Children, Glasgow

Corresponding Author: Ben C Reynolds

Dept of Paediatric Nephrology

1345 Govan Road, Glasgow, United Kingdom G51 4TF

Tel: 0141 451 6563

E-mail: ben.reynolds@ggc.scot.nhs.uk

**Supplementary Table 1**: Demographic and clinical summaries for patients 9 + 10

| **Patient** | **9** | **10** |
| --- | --- | --- |
| **Sex** | M | M |
| **Syndrome** | Pierson’s syndrome | Denys Drash Syndrome |
| **Histology** | DMS | Not done |
| **Genetic Mutation** | LAMB2 | WT1 |
| **Age at Presentation (weeks)** | 7 | 2 |
| **Initial eGFR (ml/min/1.73m^2^)** | 32.5 | 126.0 |
| **Initial Serum albumin (g/L)** | 42 | 15 |
| **Antithrombin III (IU/dL)**  **[Normal 71-101]** | 19 | 38 |
| **uPCR (g/mmol)** | 8.039 | 9.623 |
| **Enoxaparin Primary end point** | Never therapeutic, switched to warfarin after 8 weeks therapy | N/A |
| **Warfarin Primary End Point** | 19 weeks to therapeutic | N/A |
| **Outcome** | Transplant | Deceased aged 5 months |

eGFR = estimated glomerular filtration rate, uPCR = urinary protein creatinine ratio M = male, F = female, , LAMB2 = beta-2-laminin, DMS = diffuse mesangial sclerosis,

**Supplementary Table 2**: Albumin Infusion data for all included patients

| **Patient** | **Drug** | **Albumin infusion frequency** | **Albumin Infusion Dose (g/kg/week)** |
| --- | --- | --- | --- |
| **1** | Enoxaparin | Daily | 14 |
|  | Warfarin | Nil | Nil |
| **2** | Enoxaparin | Daily | 10.85 |
|  | Warfarin | Daily | 6.35 |
| **3** | Enoxaparin | Daily | 7 (initial)  22.11 (maximum at 5 weeks of LMWH)  17.77 (dose when therapeutic levels achieved) |
| **4** | Enoxaparin | Not given | Not given |
|  | Warfarin | Not given | Not given |
| **5** | Enoxaparin | Daily | 21 (initial)  31.56 (dose when therapeutic levels achieved) |
|  | Warfarin | Every 3 days | 5.16 (initial)  7.14 (dose when therapeutic levels achieved) |
| **6** | Enoxaparin | Not given | Not given |
| **7** | Enoxaparin | Daily | 7.1 (initial)  17.5 (maximum, 2 weeks of LMWH)  10 (dose when LMWH discontinued) |
| **8** | Enoxaparin | Daily | 7 (initial)  10 (dose when therapeutic levels achieved) |

LMWH = low molecular weight heparin.

**Supplementary Table 3**: Concomitant Medications at time of adverse events

| **Patient** | **Medications at time of adverse event** | **Event** |
| --- | --- | --- |
| **5** | Warfarin  IV Iron sucrose every 2 weeks  IV Darbepoetin  IV albumin 20% 3x/week with IV furosemide half-way through each infusion  Cholecalciferol  Ranitidine  Sodium chloride 30% 1mmol/ml  Levothyroxine  Enalapril  Phenoxymethylpeniciilin | Bleeding #1 |
| **5** | As above plus Esomeprazole (started after bleeding event #1) | Bleeding #2 |
| **6** | Ranitidine  Co-amoxiclav  Enoxaparin  Amlodipine  Furosemide  Cefotaxime  Morphine sulphate  Midazolam  Alimemazine  Chloral hydrate | Femoral vein thrombus |
| **8** | Levothyroxine  Phenoxymethylpenicillin  Captopril  Sodium chloride 30% 1mmol/ml | Superior vena cava thrombus |

**Clinical Vignette 1**: Anticoagulation and Complication Data for Patient 9

At 10 weeks old this patient developed a SVC thrombus whilst on dalteparin 400 units SC daily. At the time of thrombus the patient was 1 week into primary thromboprophylaxis with Anti-factor Xa level of 0.1iu/ml, eGFR of 25ml/min/1.73m^2^, serum albumin of 23g/L, uPCR of 26.56g/mmol and platelets of 83 x 10^9^/L. The patient was on continuous veno-venous haemofiltration in paediatric intensive care. An echocardiogram subsequently showed a SVC thrombus and the patient was commenced on secondary thromboprophylaxis with IV heparin 80 units/hour. This was continued until the patients transfered to the ward environment when the patient was switched to enoxaparin subcutaneously twice daily. This was targeted to an anti-factor Xa level of 0.5-1.0 iu/ml. Enoxaparin dosing was initially 2.6mg/kg/day and went on to range between 0.99-2.65mg/kg/day. The patient did not reach a therapeutic anti-factor Xa level. The patient was then commenced on 0.10mg/kg/day of warfarin with an eGFR of 11.7mg/min/1.73m^2^. The dose then ranged from 0.10-0.12mg/kg/day. After 19 weeks of therapy a therapeutic INR was reached at 0.12mg/kg/day of warfarin with an eGFR of 6.8ml/min/1.73m^2^.

eGFR = estimated glomerular filtration rate, uPCR = urinary protein creatinine ratio.

**Supplementary Figure 1:** Enoxaparin uPCR graphs

Graphs demonstrating individual patient enoxaparin dosing, therapeutic monitoring using anti-Factor Xa, eGFR and uPCR. The left y-axis displays eGFR and uPCR data, the right y-axis displays enoxaparin dose and anti-factor Xa level The grey shaded area represents the target therapeutic range for thromboprophylaxis. The vertical grey dotted line represents an adverse event.

**Supplementary Figure 2**: Warfarin uPCR graph

Graphs demonstrating patient 5 warfarin dosing dosing, therapeutic monitoring using anti-Factor Xa, eGFR and uPCR. The left y-axis displays eGFR and uPCR data, the right y-axis displays warfarin dose and anti-factor Xa level. The grey shaded area represents the target therapeutic range for thromboprophylaxis. The vertical grey dotted line represents an adverse event.

**Supplementary Figure 3**: Longitudinal data for non-included patients

1. Patient 9 parameters during enoxaparin treatment. The left y-axis displays eGFR and serum albumin data, the right y-axis displays enoxaparin dose and anti-factor Xa level.
2. Patient 9 parameters during warfarin treatment. The left y-axis displays eGFR and serum albumin data, the right y-axis displays warfarin dose and INR
3. Patient 10 parameters. The left y-axis displays eGFR and the right y-axis displays serum albumin. In all figures the grey shaded area represents the target therapeutic range for thromboprophylaxis.
